# Supplementary material for: Bioorthogonal protein-DNA conjugation methods for force spectroscopy
Source: Sci Rep. 2019 Sep 25;9:13820. doi: 10.1038/s41598-019-49843-1 (PMC6761116; doi:10.1038/s41598-019-49843-1)
Supplement: Supplementary file 1 — Supplementary Information [file 41598_2019_49843_MOESM1_ESM.pdf]

Supplementary Information

Bioorthogonal protein-DNA conjugation methods for force spectroscopy

Marie Synakewicz[1], Daniela Bauer[2], Matthias Rief[2] and Laura S. Itzhaki[1]

[1]Department of Pharmacology, University of Cambridge, Tennis Court Road, Cambridge, UK
[2]Physik-Department, Technische Universität München, James-Frank-Str. 1, Garching, Germany

Contents

1 Supporting figures **1**

2 Supporting tables **8**

3 Supporting methods **9**
3.1 Expression and purification of Sfp-synthase 9

References **9**

1 Supporting figures

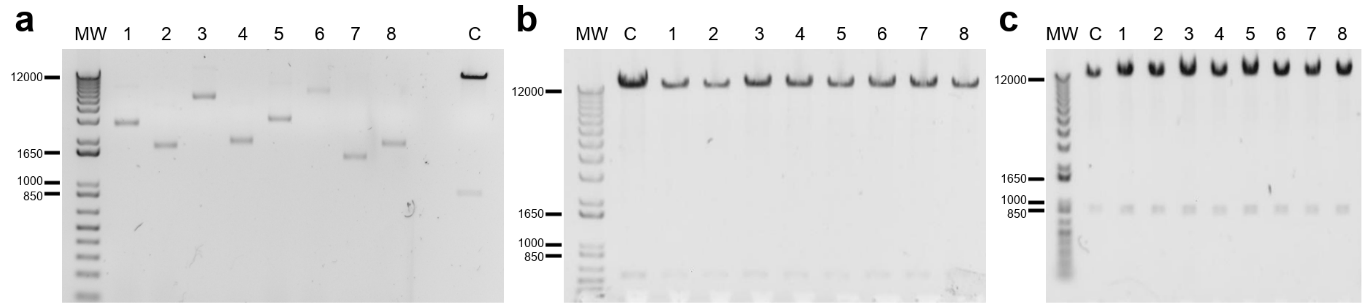

Figure S1: Transferring yPR65-277az into pRSF-oRibo-Q1-oGST using (a) SwaI/SpeI restriction cloning, (b) the Fastclone method [1], or (c) In-Fusion cloning. Shown are BamHI restriction digests of plasmids derived from 8 bacterial colonies each, together with a digest of a control plasmid (C, pRSF-oRibo-Q1-oGST-$PR65_{5/588TAG}$). The frequency of obtaining a correct plasmid using restriction cloning was very low and many recombination events were observed. Only pRSF-oRibo-Q1-oGST-$PR65_{5/588TAG}$ was obtained using this method. The Fastclone method did not yield any correct clones, whereas In-Fusion cloning normally produces the required construct in 80-100% of the cases. MW - molecular weight marker (Invitrogen 1kb Plus).

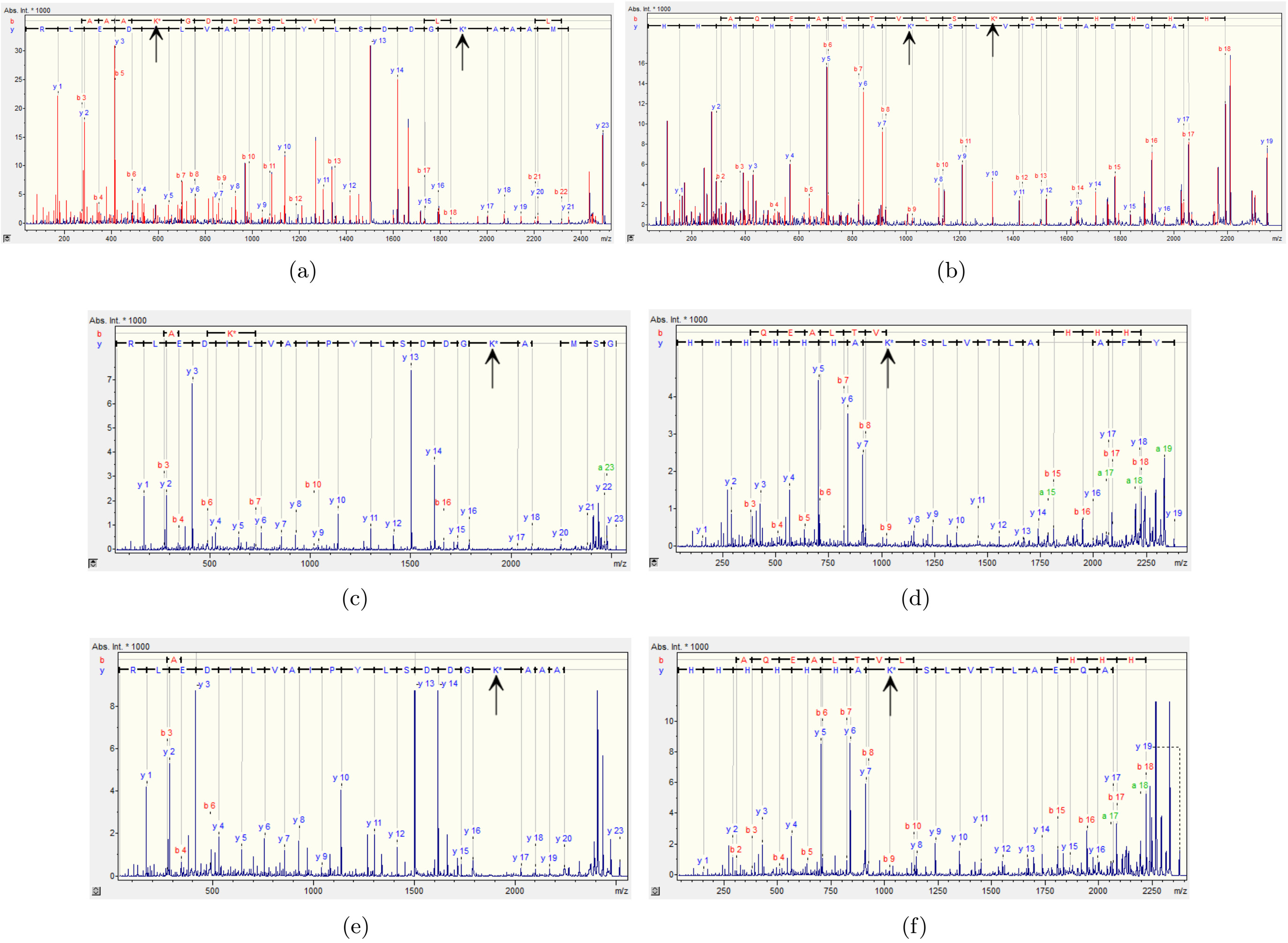

Figure S2: Tandem mass-spectrometry of PR65 peptides with UAAs incorporated at positions 5 and 588 (arrows). Shown are the N-/C-terminal peptides of alkPR65 (a,b), azPR65 (c,d), and cycPR65 (e,f). The figure was provided by the PNAC Facility of the Biochemistry Department, University of Cambridge.

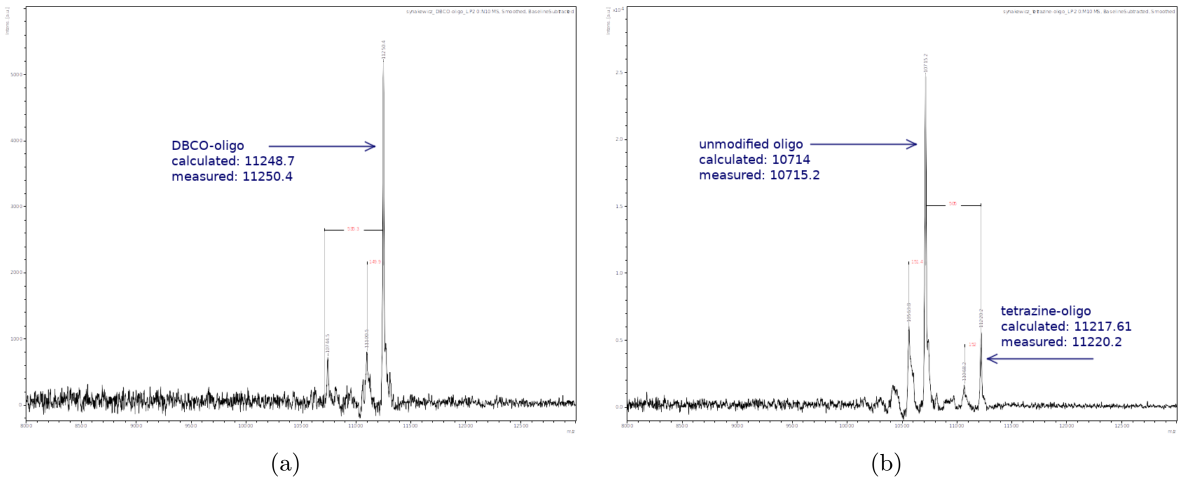

Figure S3: MALDI mass-spectrometry of modified oligonucleotides (a) DBCO-oligo (b) tetrazine oligo. The figure was provided by the PNAC Facility of the Biochemistry Department, University of Cambridge.

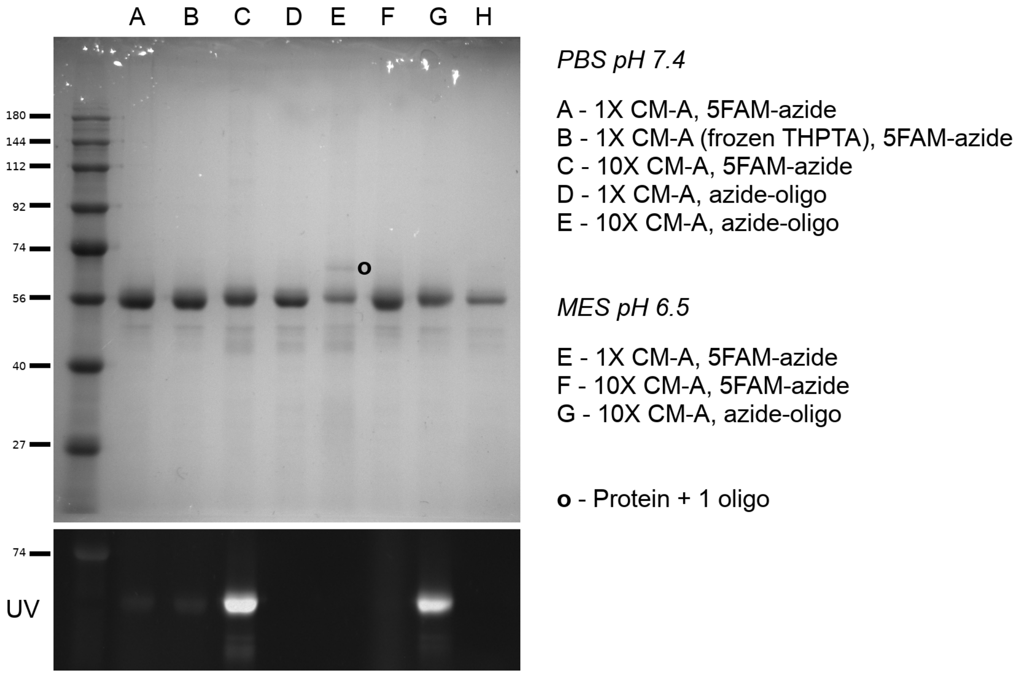

Figure S4: Example of click reactions using 100 µM 5-FAM azide or 3'-azide DNA oligo and 5 µ $PR65_{5/588}$ bearing alkyne moieties. Reactions were performed in PBS or MES buffer. Click-mix concentrations are given in fold excess of the standard CM-A concentration (click-mix containing 250 mM NaAsc).

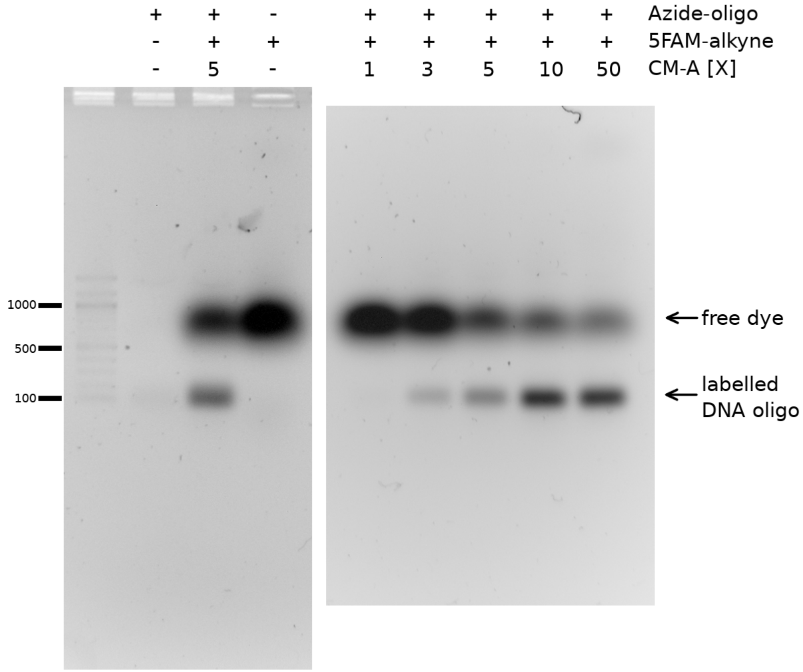

Figure S5: Click reactions of azide-oligo and 5-FAM-alkyne with increasing concentrations of CM-A (click-mix containing 250 mM NaAsc) on an unstained 1% agarose gel. Click-mix concentrations are given in fold excess of CM-A.

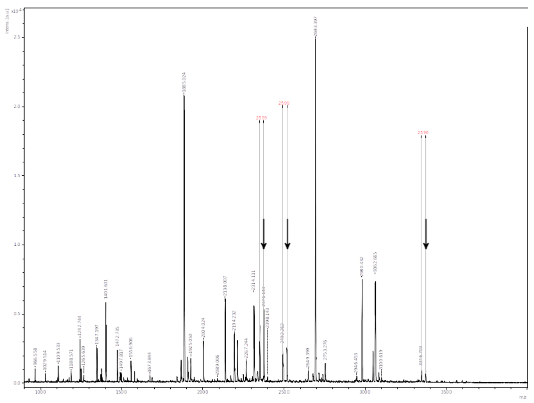

Figure S6: Each peptide containing azido-propargyllysine (arrows) is accompanied by a similar-size signal of -25.99amu resulting from the reduction of azide to amine. The figure was provided by the PNAC Facility of the Biochemistry Department, University of Cambridge.

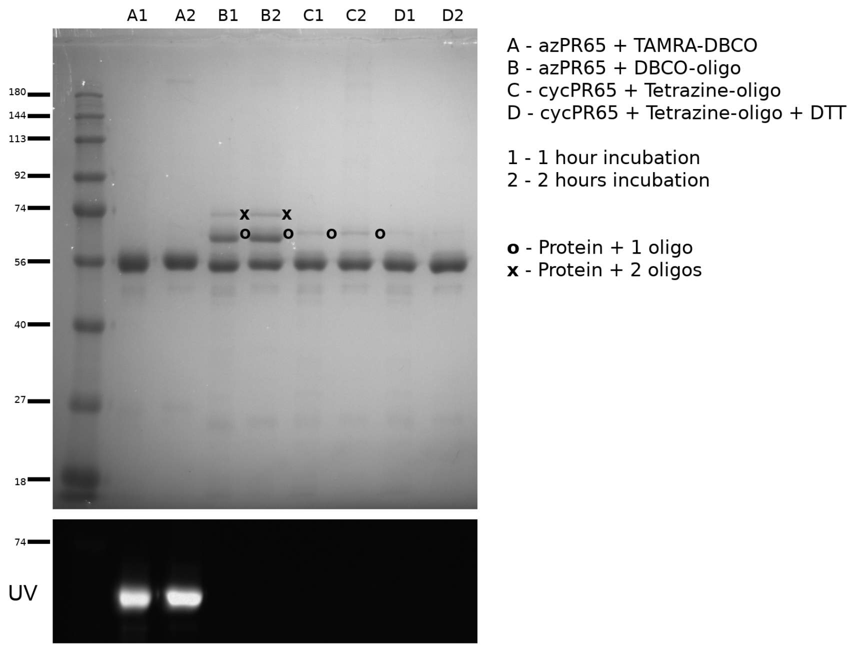

(a)

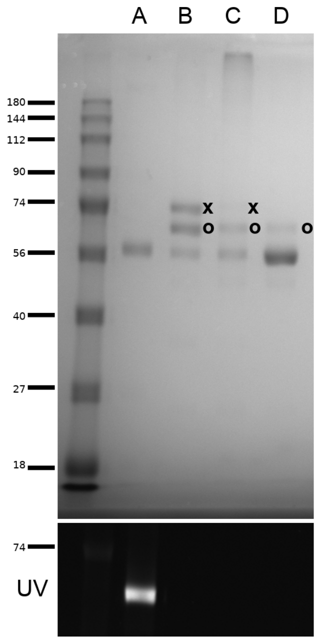

(b)

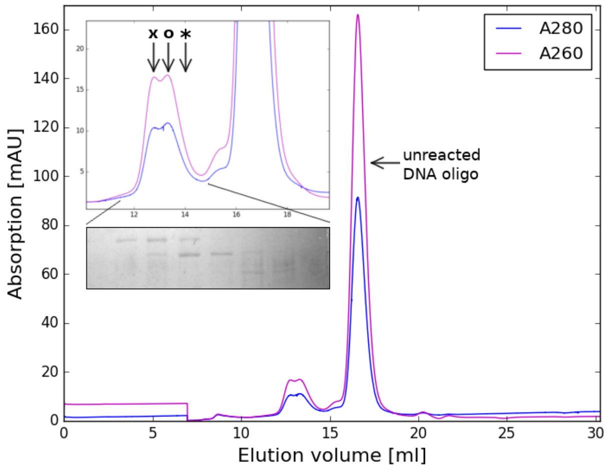

(c)

Figure S7: Full gels of Figure 3 in the main paper. SPAAC and IED-DA between azide or cyclopropene containing PR65$_{5/588\mathrm{TAG}}$ and TAMRA or oligo. (a) Example reactions using 5 µM protein and 20 µM TAMRA or oligo (b) Same reactions as in (a), incubated over night. (c) Chromatogram of a large scale reaction of 10 µM azPR65az and 40 µM DBCO-oligo incubated over night at 25°C, separated on a S200 10/300 GL. The smaller peak doublet preceding the oligo peak corresponds protein conjugated to two oligos (**x**) and one oligo (**o**). Un-reacted protein can be detected by SDS-PAGE even though a clear A280 peak is not visible (*****, inset).

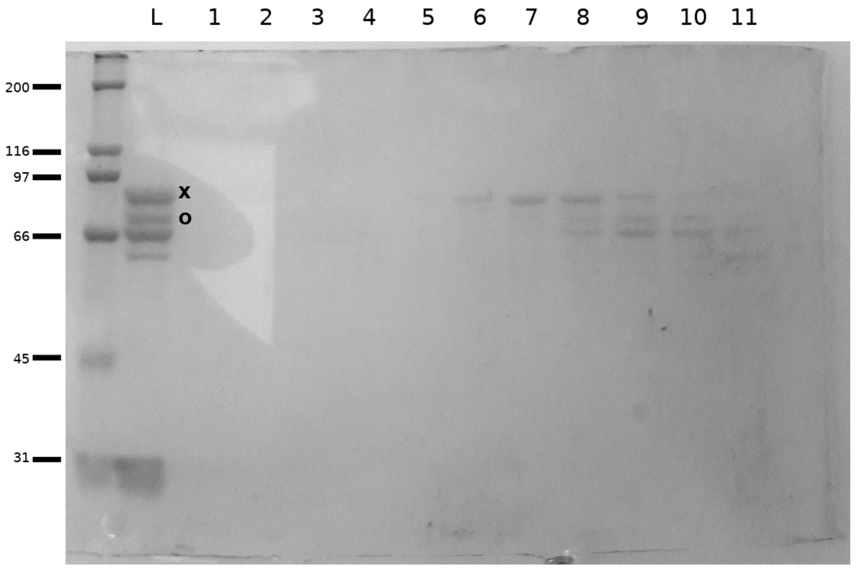

Figure S8: Sfp-synthase mediated attachment of 20 µM CoA-oligos to 10 µM of protein bearing N- and C-terminal ybbR-tags after an over-night incubation at room temperature (L). Lanes 1-11 are consecutive elution fractions after HPLC. Protein conjugated to two oligos (**x**) and one oligo (**o**) are present.

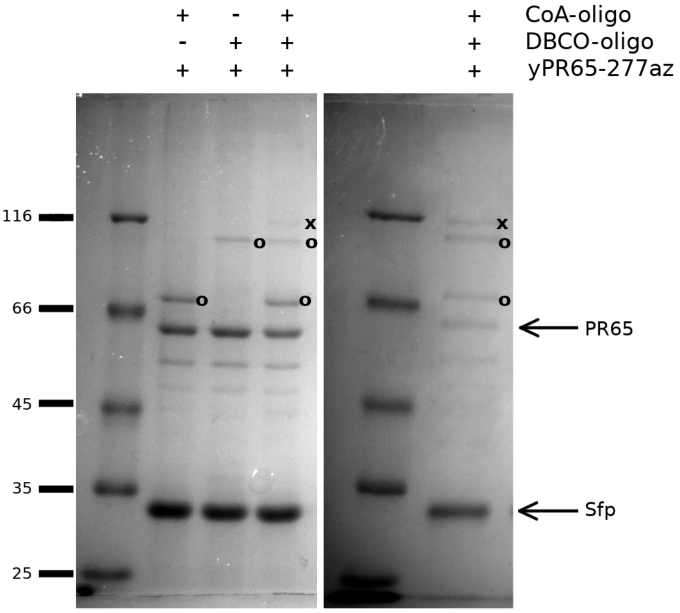

Figure S9: Combining CoA-ybbR and SPAAC conjugations. Conjugations of yPR65-277az to DBCO- and CoA oligonucleotides after 2 hours (left) and over-night incubation (right).

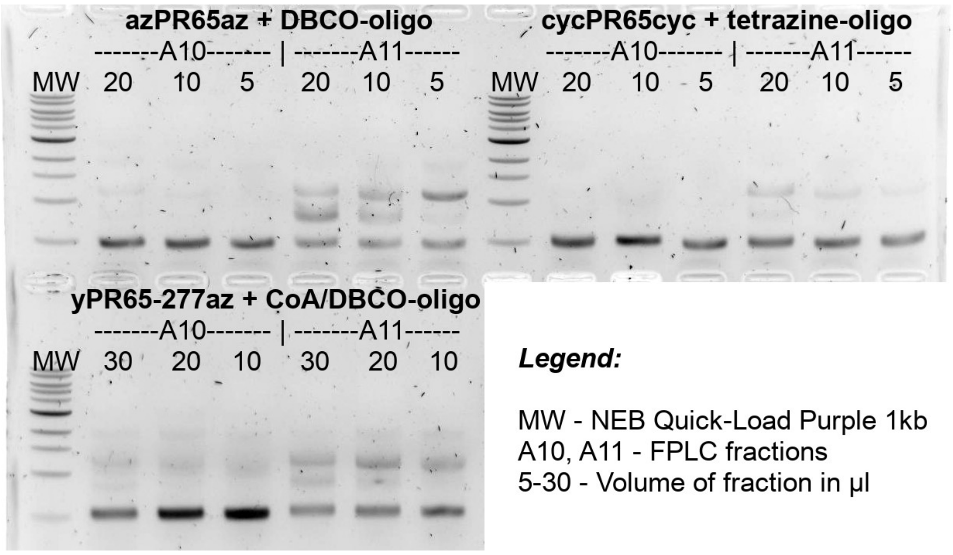

Figure S10: DNA handle hybridization to SPAAC, IE-DA and Sfp-mediated protein-oligo conjugations purified using an S200 10/300 GL column and detected by agarose gel electrophoresis. Shown are hybridization products between varying amounts of protein from two different fractions to double stranded DNA handles.

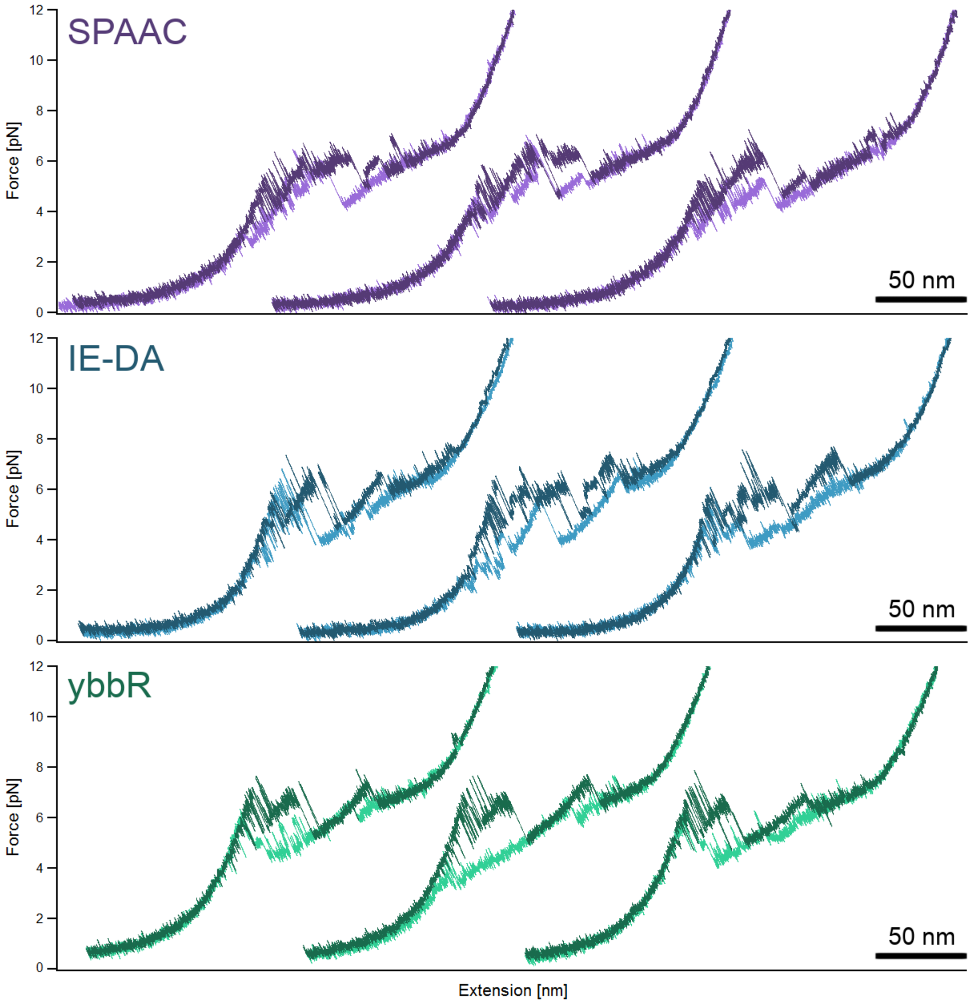

Figure S11: Representative full-length force extension curves of the same molecule for each PR65 attachments. Unfolding traces are coloured in darker shades, while refolding traces are coloured in lighter shades. All traces were taken at pulling speeds of 10 nm s^{-1}.

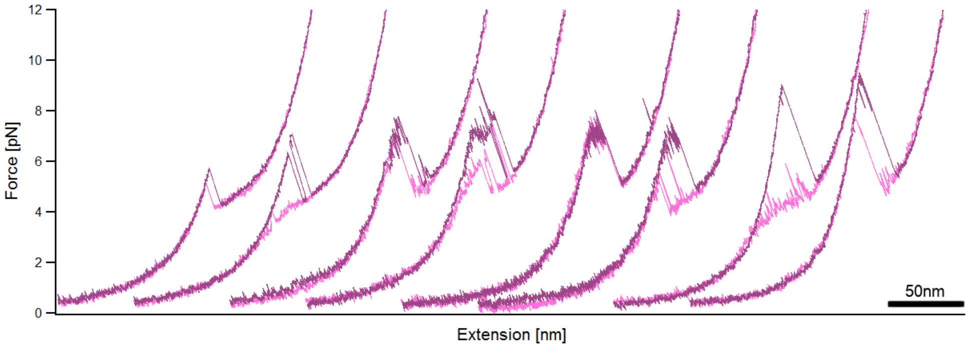

Figure S12: Two representative stretch-relax cycles of four yPR65-277az molecules. All data shown were obtained at pulling velocities of 10 nm s^{-1}.

2 Supporting tables

Table S1: Equilibrium denaturation three-state fit parameters of PR65 variants. The parameters represent averages ± s.e.m. of three technical replicates.

Protein	$D_{50\%-1}$ [M]	m_1 [kcal mol^{-1} M^{-1}]	$D_{50\%-2}$ [M]	m_2 [kcal mol^{-1} M^{-1}]
PR65	2.24 ± 0.06	2.51 ± 0.09	5.13 ± 0.03	1.40 ± 0.02
yPR65y	2.14 ± 0.06	2.48 ± 0.06	5.20 ± 0.05	1.16 ± 0.06

Table S2: List of DNA oligonucleotides (5' to 3'). * indicates sites of modification

Name	Sequence
PR65 D5TAG Fwd	TAGGGCGACGACTCGCTGTACCCC
PR65 D5TAG Rev	GGCCGCCGCCATATGATGATGATGG
PR65 L588TAG Fwd	TAGGCCCACCACCACCACCACC
PR65 L588TAG Rev	AGACAGAACAGTCAGAGCCTCCTGGGC
PR65 E277 Fwd	TAGATCACCAAGACAGACCTGGTC
PR65 E277 Rev	AGGCCCCACTGCTTTCTGG
oRibo Inf SwaI Fwd	TCATAAAACATATTTAAATGGTGATCATGTAACCCATCC
oRibo Inf SpeI Rev	GTACGGCCGACTAGTTCAGTGGTGGTG GTGGTGGTGGGCGAGAG
PR65 NybbR Fwd	GATTCTCTTGAATTTATTGCTAGTAAGC TTGCGATGGCGGCGGCCGACGGCGACG
PR65 NybbR Rev	GGATCCACGCGGAACCAGATCCGATTTTGG
PR65 CybbR Fwd	CACCACCACCACCACCACTGAAAGC
PR65 CybbR Rev	CGCAAGCTTACTAGCAATAAATTCAAGAG AATCGGCGAGAGACAGAACAGTCAGAGC
DNA-protein coupling	GGCAGGGCTGACGTTCAACCAGACCAGCGAGTCG*
Biotin/digoxigenin	*GGCGA*CTGG*CGTTGATTTG
Abasic	CGACTCGCTGGTCTGGTTGAACGTCAGCCC TGCC*CCTGCCCGGCTCTGGACAGG

3 Supporting methods

3.1 Expression and purification of Sfp-synthase

The pCK plasmid containing Sfp-synthase-H_6 was a kind gift from the Gaub laboratory (Ludwig Maximilian Universität, München, Germany). The previously published protocol for the production of Sf-synthase [2] was adapted as follows: For expression, the plasmid was transformed into chemically competent C41 *E. coli* and plated onto LB Agar containing Ampicillin. All colonies from the plate were used to inoculate 1 l of 2xYT. Protein expression was induced at $OD_{600} \sim 0.6$ by the addition of 1 mM IPTG and followed by further incubation at 25°C overnight. Following centrifugation, the pellet was resuspended in lysis buffer (20 mM Tris-HCl pH 7.5, 500 mM NaCl, 5 mM imidazole) containing protease inhibitor cocktail and DnaseI, lysed as described above and centrifuged again to remove cell debris. The soluble protein fraction was filtered through a 0.22 µm PES membrane and applied to a 5 ml HisTrap Excel column (GE Healthcare) equilibrated in lysis buffer. After washing with 20 column volumes of buffer, Sfp was eluted in one step using 20 mM Tris-HCl pH 8.0, 300 mM imidazole, 300 mM NaCl, 2 mM EDTA. The elution fractions were analysed by SDS-PAGE and those containing protein at >90% purity were pooled. This solution was then split in half and dialysed twice against either 10 mM Tris-HCl pH 7.5 or PBS pH 7.4 containing 1 mM EDTA and 10% (v/v) glycerol. Protein was flash-frozen after concentration using a Vivaspin®centrifugal concentrator and stored at -80°C.

References

[1] C. Li, A. Wen, B. Shen, J. Lu, Y. Huang, and Y. Chang. FastCloning: a highly simplified, purification-free, sequence- and ligation-independent PCR cloning method. *BMC Biotechnology*, 11(92), 2011.

[2] J. Yin, A. J. Lin, D. E. Golan, and C. T. Walsh. Site-specific protein labeling by Sfp phosphopantetheinyl transferase. *Nature Protocols*, 1:280, 2006.
